# Multifocal and Microvascular Involvement in Ischemic Stroke During COVID-19: A Cohort Study With Comparison With Non-COVID-19 Stroke

**DOI:** 10.3389/fneur.2021.732194

**Published:** 2021-10-25

**Authors:** Geoffroy Hautecloque, Christian Kempf, Camélia Stan, Marie-Hélène Arentz-Dugay, Francis Vuillemet, Guido Ahle, François Sellal, Martin Martinot

**Affiliations:** ^1^Department of Neurology, Hôpitaux Civils de Colmar, Colmar, France; ^2^Clinical Research Department, Hôpitaux Civils de Colmar, Colmar, France; ^3^Inserm U-1118, Strasbourg University, Strasbourg, France; ^4^Department of Infectious Diseases, Hôpitaux Civils de Colmar, Colmar, France

**Keywords:** COVID-19, coagulopathy, inflammation, cerebrovascular, observational study, ischemic stroke

## Abstract

**Introduction:** Thromboembolic events, including ischemic stroke, are major complications of coronavirus disease 2019 (COVID-19). The clinical characteristics of COVID-19-related stroke are not clearly defined, and few controlled studies assessed the underlying mechanisms of cerebrovascular complications of COVID-19. This single-center retrospective observational study compared stroke characteristics between patients with and without COVID-19.

**Methods:** This study included all patients hospitalized between March 1, 2020, and April 30, 2020, in Colmar Hospital for ischemic stroke as confirmed by imaging. The characteristics of patients with laboratory-confirmed severe acute respiratory syndrome coronavirus-2 (SARS-CoV-2) infection by real-time reverse transcriptase polymerase chain reaction or serology were compared with those without SARS-CoV-2 infection.

**Result:** Among 772 patients, nine COVID-19 patients were compared with 50 patients without COVID-19. The following inflammatory and procoagulant marker levels were significantly higher in the COVID-19 group than those in the control group: C-reactive protein, 57.3 ± 43.4 vs. 15.0 ± 30.6 mg/L, *p* < 0.001; fibrinogen, 5.89 ± 1.75 vs. 4.03 ± 1.26 g/L, *p* < 0.001; and D-dimer, 4,833.9 ± 6,549.4 vs. 1,028.6 ± 942.6 ng/ml, *p* < 0.001. The rates of multifocal cerebral territory involvement (4 vs. 7, *p* = 0.05), microvascular involvement (4 vs. 6, *p* = 0.04), and thrombophilia (4 vs. 4, *p* = 0.014) were significantly higher in the COVID-19 group than in the control group, whereas no significant intergroup differences were found in the stroke mechanisms, i.e., cardio-embolic, atherosclerotic, small vessel disease, and cryptogenic.

**Conclusion:** COVID-19-related stroke is characterized by hypercoagulability and hyperinflammation that may favor strokes via microvascular circulation abnormalities, microthrombus formation, and multifocal lesions.

## Introduction

Coronavirus disease 2019 (COVID-19) is a polymorph disease caused by the novel severe acute respiratory syndrome coronavirus-2 (SARS-CoV-2) that emerged in December 2019 in China and rapidly spread worldwide. COVID-19 primarily affects the respiratory tract, with clinical presentation ranging from paucisymptomatic upper respiratory tract infection to systemic acute respiratory syndrome (1). Thromboembolic events have emerged as major manifestations of COVID-19, occurring in 7.7–30.0% of patients (2, 3), and ischemic stroke is a prominent thromboembolic complication of COVID-19, with an incidence ranging from 1.1 to 5% (4–6) among hospitalized patients with COVID-19. Although poorly understood, multiple factors, including disseminated intravascular coagulation, hypercoagulability, thrombophilia with anticardiolipin or anti-β2GP1 antibodies, cardiac injury, and arrhythmia, have been proposed in the etiology of stroke in patients with COVID-19 (7–9).

Colmar hospital is a 1,000-bed facility, and the main hospital of center Alsace GHT11 has a unique stroke unit for its territory of 410,000 inhabitants. In March 2020, Alsace in Northeastern France was hit by a major outbreak of COVID-19, and Hôpital Civil de Colmar was one of the most affected hospitals by the pandemic. In this single-center retrospective study, we assessed risk factors for ischemic stroke, including transient ischemic stroke, in patients with COVID-19 and elucidated the underlying mechanisms by comparing the characteristics of patients with laboratory-confirmed COVID-19 who experienced stroke with those of patients hospitalized for stroke without COVID-19 during the same period in our hospital.

## Materials and Methods

### Inclusion and Exclusion Criteria

**Figure 1:**
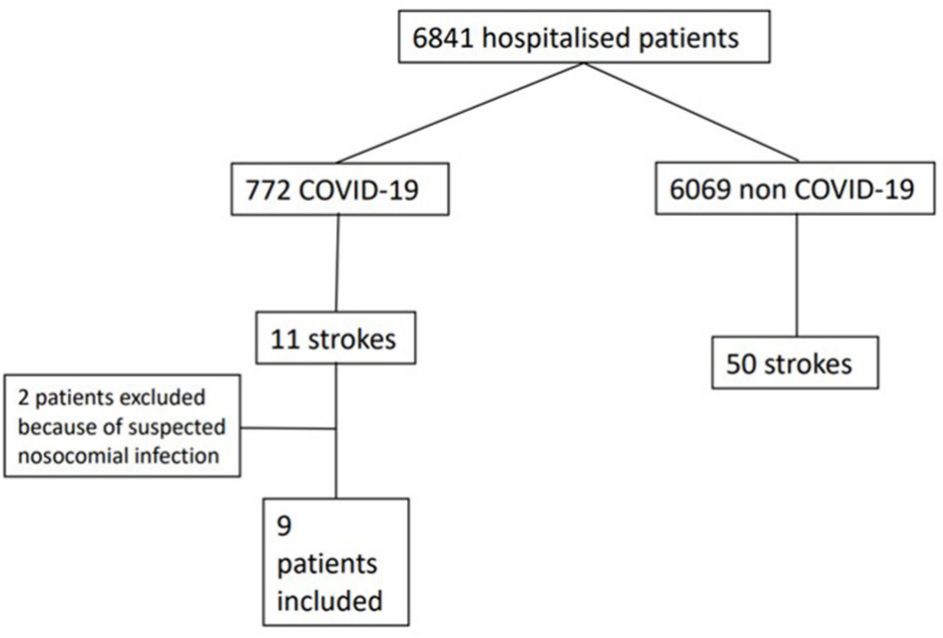
Flowchart.

The study included all patients hospitalized between March 1, 2020, and April 30, 2020, in Hôpital Civil de Colmar and had acute ischemic stroke or transient ischemic attack (TIA) upon admission or during hospitalization. Acute ischemic stroke has been defined as acute neurological dysfunction caused by an ischemic injury confirmed by the presence of cytotoxic edema on brain magnetic resonance imaging (MRI) (increased diffusion-weighted imaging signal and reduction of apparent diffusion coefficient). T2/fluid-attenuated inversion recovery (FLAIR), time-of-flight (TOF), and susceptibility-weighted imaging (SWI) sequences had also been performed to rule out differential diagnoses and to search for arterial occlusion when visualizable. When MRI could not be realized owing to the presence of a pacemaker, ischemic stroke was confirmed by the presence of a low density on computed tomography (CT). TIA was defined as a transient episode of neurological dysfunction caused by focal brain or retinal ischemia, without acute infarction (10). The COVID-19 group included patients with positive RT-PCR within 48 h of admission. The control group included patients hospitalized for ischemic stroke or TIA without COVID-19, i.e., negative for reverse transcriptase polymerase chain reaction (RT-PCR) or for serology performed during hospitalization. Patients who were hospitalized for stroke and were positive for SARS-CoV-2 by nasopharyngeal swab or sputum test 48 h after admission were excluded from the study, because they were suspected of contracting the infection during hospitalization, and those admitted with clinical signs of COVID-19 without confirmation by real-time RT-PCR or serology (Figure 1) were also excluded.

### Stroke Classification

In the present study, the causes of stroke were categorized according to the Trial of ORG 10172 in Acute Stroke Treatment (TOAST) classification (11) and the embolic stroke of undetermined source criteria (12). Ischemic stroke due to atherosclerotic disease was defined as ≥50% narrowing in a large artery due to an atheroma plaque or intracranial stenosis of an artery perfusing the ischemic infarct territory. Involvement of a large artery was defined as the thrombotic occlusion in vertebral artery, basilar artery, first segment of posterior cerebral artery carotid artery, first and second segments of middle cerebral artery, or first portion of anterior cerebral artery. Stroke due to arteriogenic emboli (aortic arch atherosclerotic plaques or non-stenotic cerebral artery plaques with ulceration) were also considered as atherosclerotic causes of ischemic stroke. Stroke due to small vessel disease was defined as a small subcortical infarct (≤ 2 cm on diffusion-weighted imaging sequence or ≤ 1.5 cm on CT) in patients with cardiovascular risk factors for small vessel disease. Cardio-embolic infarct was defined based on the presence of a cardio-embolic source, such as atrial fibrillation or atrial dysrhythmia and stasis, patent foramen ovale with atrial septal aneurysm in patients aged <60 years, with no other obvious cause, or left ventricular dysfunction. Strokes associated with myocardial infarct/takotsubo cardiomyopathy/myocarditis were also considered to arise from a cardio-embolic source. Other etiologies included alternative mechanisms such as dissection or procoagulant state such as hereditary thrombophilia (activated protein C deficiency). Strokes without an overt etiology were classified as cryptogenic.

### Data Collection

The following data were collected from the electronic medical records of patients: (1) demographic characteristics (age and sex); (2) comorbidities/high-risk conditions (history of hypertension, dyslipidemia, diabetes, obesity and high body mass index, smoking, atrial fibrillation, obstructive sleep apnea, peripheral artery disease, supra-aortic trunk stenosis, cardiopathy, stroke, and inflammatory disease); (3) stroke characteristics (TIA or stroke, National Institutes of Health Stroke Scale [NIHSS] score, large-artery occlusion, multi-territorial location, microvascular lesion [ <5 mm in size], time between the first COVID-19 symptoms and stroke, and stroke etiology); (4) laboratory data (C-reactive protein, coagulation parameters [D-dimer, fibrinogen, lupus anticoagulant, anticardiolipin antibody, and anti-β2 glycoprotein-1 antibody]); and (5) severity of COVID-19 infection (mild, moderate, or severe) (13).

### Statistical Analysis

The patient baseline characteristics of the overall cohort and the COVID-19 and control groups were analyzed. Continuous variables were summarized as means with standard deviation (SD) and compared using Student's *t*-test or Wilcoxon's rank-sum test when the hypothesis of homoscedasticity was not satisfied. Categorical data were presented with numbers of missing values and absolute and relative counts and compared using the chi-squared test or Fisher's exact test when conditions were not satisfied for the chi-squared test. Given the retrospective, descriptive nature of the study, test results were given as an indication. No corrections for multiplicity were performed. All statistical analyses were performed using SAS 9.4 (SAS Institute, Cary, NC, USA).

The study was approved by the Comité d'Ethique des Facultés et Hôpitaux de Médecine, Odontologie et Pharmacie of the University Hospital of Strasbourg (approval no. CE-2020-32).

## Results

Among a total of 6,841 patients hospitalized between March 1, 2020, and April 30, 2020, in Hôpital Civil de Colmar, 772 patients have been identified with SARS-CoV-2 infection, of whom 11 patients had a stroke. Two patients whose COVID-19 symptoms emerged more than 48 h (3 and 19 days) after hospitalization for stroke were excluded from the study. In total, nine patients (1.16%) were included, i.e., eight with ischemic stroke and one with TIA (Table 1). During the same period, among 6,069 patients without past or concomitant history of COVID-19 hospitalization, 43 patients were admitted for stroke and seven for TIA. In the control group, no clinical symptoms of COVID-19 have been observed, and RT-PCR was performed in 17 (34%) patients and serology in nine (18%). The stroke diagnosis was based on MRI in 60 of the patients (98.4%), whereas stroke was diagnosed based on CT in one patient (1.6%) with a pacemaker. All patients had intracranial and extracranial vascular imaging and cardiac evaluation including ECG, in-house continuous cardiac telemetry for at least 24 h, and transthoracic echocardiography. Young patients (age, <60 years) without obvious causes were screened for thrombophilia. Transesophageal ultrasound was performed in patients suspected of having a patent foramen ovale.

**Table 1:** Demographic, clinical, biological, and radiological data and mechanism of the nine COVID-19 patients.

**Patient**	**Sex and age (years)**	**RT-PCR**	**COVID19 severity**	**CRP (mg/L)**	**NIHSS**	**Clinical manifestation of stroke**	**Time between stroke and COVID-19 onset (days)**	**Past medical history**
1	M, 78	+	Moderate	22	1	Transitory aphasia	−1	Past smoking, diabetes
2	W, 71	+	Moderate	105	7	Left facial paresis, left hemihypoesthesia, dysarthria, heminegligence	0	HTA, active smoking, obstructive sleep apnea, atrial fibrillation, metastatic ovarian carcinoma (discover 2 month after COVID19 infection)
3	M, 59	+	Moderate	4	13	Left hemiplegia, left hemihypoesthesia	−1	HTA, active smoking, obesity
4	M, 78	+	Moderate	53	6	Dysarthria, right proportional hemiparesis	14	HTA, psoriasis, hypothyroidism
5	M, 53	+	Severe	91	2	Diplopia	19	Past smoking, obesity, ankylosing spondylitis, protein C deficit
6	M, 72	+	Severe	30	2	Temporospatial disorientation, incoherent speech	24	Dyslipidemia, diabetes, past smoking, obstructive sleep apnea, paroxysmal atrial fibrillation, obesity, asthma
7	M, 66	+	Severe	75	16	Left upper limb paresis, left facial paresis, dysarthria	4	HTA, dyslipidemia, diabetes, supra-aortic trunks stenosis (55–60% right carotid artery stenosis, 60% right vertebral artery stenosis, >90% left vertebral artery stenosis), ischemic cardiopathy, gout, mild renal chronic insufficiency, alcohol consumption
8	W, 86	+	Severe	12	10	Left hemiplegia, left hypoesthesia	16	HTA, dyslipidemia, diabetes, stroke, obesity, supra-aortic trunks stenosis (50% bilateral carotid artery stenosis), valvular cardiopathy, non-secreting suprarenal tumor
9	M, 62	+	Critical	124	–	Difficulty waking up	18	Dyslipidemia, diabetes, obesity, hypothyroidism
**Patient**	**Thrombophillia screening**	**Microvascular involvement (<0.5 cm)**	**Multi-territorial involvement**	**Location**	**Mechanism**	**Thrombolysis**	**Fg (g/L)**	**D-Dimer (ng/mL)**
1	0	No	No	No new lesion	Cryptogenic	No	5.2	560
2	Lupus anticoagulant	No	No	Right superficial middle cerebral artery	Cardio-embolic (atrial fibrillation)	No	2.85	>20,000
3	Lupus anticoagulant	No	No	Right superficial middle cerebral artery	Cryptogenic	Yes	4.87	2,868
4	0	Yes	Yes	Right superficial middle cerebral artery + microvascular left superficial middle cerebral artery	Cardio-embolic (burst of atrial tachycardia)	No	7.84	–
5	Protein C deficit, Lupus anticoagulant	No	No	Right deep posterior cerebral artery	Thrombophilia (protein C deficit)	Yes	8.54	466
6	0	Yes	Yes	Right superficial middle cerebral artery + one small left superficial middle cerebral artery cortical infarct + one small right cerebellar artery infarct	Cardio-embolic (atrial fibrillation)	No	6.01	993
7	Lupus anticoagulant	Yes	Yes	Right superficial middle cerebral artery + one small left superficial middle cerebral artery cortical infarct	Cardio-embolic (concomitant cardiac infarct)	Yes	5.87	5,243
8	0	No	No	Right deep middle cerebral artery	Atheromatosis	No	4.72	6,867
9	0	Yes	Yes	Bilateral superficial middle cerebral artery + anterior cerebral artery	Cryptogenic	No	7.12	1,674

*CRP, C-reactive protein; Fg, fibrinogen; HTA, hypertension; ICU, intensive care unit; M, man; RT-PCR, reverse transcriptase polymerase chain reaction; W, woman; COVID-19, coronavirus disease 2019*.

The median ages were 69.4 ± 10.5 and 70.6 ± 13.2 years in the COVID-19 and control groups, respectively. Male patients comprised 77.8 and 70% of the COVID-19 and control groups, respectively. The percentages of patients with specific high-risk conditions are presented in Table 2. Only one of the nine patients with COVID-19 was admitted to the intensive care unit (ICU). The mean time interval between the first COVID-19 symptoms and the first stroke symptoms was 10.3 ± 9.8 days. TIAs occurred in one patient (11.1%) in the COVID-19 group and six patients (12.0%) in the control group (*p* > 0.999; Table 2).

**Table 2:** Demographic, clinical, biological, and radiological data and mechanism in COVID-19 and control group.

		**Non-COVID-19**	**COVID-19**	**Total**	***p*-value**
		**(*N* = 50)**	**(*N* = 9)**	**(*N* = 59)**	
Sex, Male		35 (70%)	7 (77.8%)	42 (71.2%)	NS
Age (years)	*N* (%)	50 (100.00%)	9 (100.00%)	59 (100.00%)	NS
	Mean (SD)	70.6 (13.2)	69.4 (10.5)	70.4 (12.8)	
	Range	[43–92]	[53–86]	[43–92]	
BMI (kg/m^2^)	*N* (%)	42 (84%)	9 (100.00%)	51 (86.44%)	NS
	Mean (SD)	26.26 (4.84)	29.24 (4.96)	26.78 (4.95)	
	Range	[17.8–38.8]	[21.3–36.3]	[17.8–38.8]	
Medical history		48 (94.1%)	9 (100.0%)	57 (95.0%)	NS
Hypertension		29 (58%)	5 (55.6%)	34 (57.63%)	NS
Dyslipidaemia		19 (38%)	4 (44.4%)	23 (38.98%)	NS
Diabetes mellitus		17 (34%)	3 (33.3%)	20 (33.9%)	NS
Smoking habits		23 (46%)	5 (55.56%)	28 (47.46%)	NS
Atrial fibrillation		12 (24%)	2 (22.2%)	14 (23.7%)	NS
Obstructive sleep apnea		7 (14%)	0 (0%)	7 (11.9%)	NS
Stroke		6 (12%)	1 (11.1%)	7 (11.9%)	NS
Obesity		12 (27.2%)	5 (55.6%)	17 (32.1%)	NS
Supra-aortic trunks stenosis		16 (32%)	2 (22.2%)	18 (30.5%)	NS
Cardiopathy		8 (16%)	1 (11.1%)	9 (15.3%)	NS
PAD		4 (8%)	-	4 (6.8%)	NS
Inflammatory disease		10 (20%)	2 (22.2%)	12 (20.3%)	NS
Other		33 (66%)	7 (77.8%)	40 (67.8%)	NS
CRP (mg/L)	*N* (%)	49 (98.0%)	9 (100.00%)	58 (98.31%)	<0.001^a^
	Mean (SD)	15.0 (30.6)	57.3 (43.4)	21.57 (35.95)	
	Range	[0–124]	[4-124]	[0–124]	
D-Dimer	*N* (%)	42 (84%)	8 (88.89%)	50 (84.75%)	<0.001^a^
	Mean (SD)	1,028.6 (942.6)	4,833.9 (6,549.4)	1,637.48 (2,976)	
	Range	[139–3,795]	[466–20,000]	[139–20,000]	
Fibrinogen	*N* (%)	48 (96%)	9 (100.00%)	57 (96.61%)	<0.001^a^
	Mean (SD)	4.03 (1.26)	5.89 (1.75)	4.32 (1.50)	
	Range	[2.3;7.9]	[2.9;8.5]	[2.3;8.5]	
Time since first symptoms	*N* (%)		9 (100.00%)	9 (15.00%)	
	Mean (SD)		10.3 (9.8)	10.3 (9.8)	
	Range		[−1;24]	[−1;24]	
Transient ischemic attack		6 (12%)	1 (11.1%)	7 (11.86%)	NS
NIHSS	*N* (%)	50 (100.00%)	9 (100.00%)	59 (100.00%)	NS
	Mean (SD)	4.8 (5.8)	6.3 (5.7)	5.03 (5.8)	
	Range	[0–20]	[0–16]	[0–20]	
Etiology Cardioembolic		16 (32%)	4 (44.4%)	20 (33.9%)	NS
Atheroma and small vessel disease		21 (42%)	1 (11.1%)	22 (37.3%)	NS
Other		2 (4%)	1 (11.1%)	3 (5.1%)	NS
Cryptogenic		11 (22%)	3 (33.3%)	14 (23.7%)	NS
Thrombophilia screening		3 (6.0%)	4 (44.44%)	7 (11.9%)	0.008^b^
Multi-territorial infarction		7 (14%)	4 (44.4%)	11 (18.64%)	0.05^b^
Large-artery occlusion		10 (20%)	3 (33.3%)	13 (22.03%)	NS
Death		5 (10%)	0 (0%)	5 (8.47%)	NS
Microvascular lesion (<0.5 cm)		6 (12%)	4 (44.44%)	13 (22.03)	0.04^b^
Mechanical thrombectomy and/or thrombolysis		10 (20%)	4 (44.44%)	14 (23.73%)	NS

*COVID-19, coronavirus disease 2019; BMI, body mass index; CRP, C-reactive protein; NIHSS, National Institutes of Health Stroke Scale; NS, nonsignificant; PAD, peripheral artery disease; SD, standard deviation*.

a *Student t-test*.

b *Fisher exact test*.

The levels of laboratory markers for inflammation and thrombosis at the time of stroke were higher in the COVID-19 group compared with the control group (C-reactive protein, 57.3 ± 43.4 vs. 15.0 ± 30.6 mg/L, *p* < 0.001; fibrinogen, 5.89 ± 1.75 vs. 4.03 ± 1.26 g/L, *p* < 0.001; and D-dimer, 4,833.9 ± 6,549.4 vs. 1,028.6 ± 942.6 ng/ml, *p* < 0.001). The frequency of patients with positive thrombophilia parameters was higher in the COVID-19 group than in the control group (4/9 [44.4%] vs. 3/50 [6%]; *p* = 0.008). All seven patients were positive for lupus anticoagulant.

No clinical signs have been observed suspicious of a venous thromboembolic event among our COVID-19 patients.

The causes of stroke were not significantly different between the COVID-19 and control groups. Cardio-embolic factors were a stroke cause in four (44.4%) patients in the COVID-19 group and 16 (32.0%) patients in the control group (*p* = 0.47). Although not statistically significant, atherosclerosis and small vessel disease were less frequent causes of stroke in the COVID-19 group (1/9 [11.1%]) than in the control group (21/50 [42.0%]) (*p* = 0.13). Cryptogenic stroke was diagnosed in three (33.3%) patients in the COVID-19 group and 11 patients (22%) in the control group (*p* = 0.43). In the COVID-19 group, the only other etiology was found in a patient with a history of thrombophilia who was diagnosed with activated protein C deficiency several years before the stroke. In the control group, stroke was provoked by arterial dissection and in the context of neoplasia in each patient.

The stroke severity based on the NIHSS score was comparable between the two groups (6.3 [5.7] and 4.8 [5.8] in the COVID-19 and control groups, respectively; *p* = 0.467).

**Figure 2:**
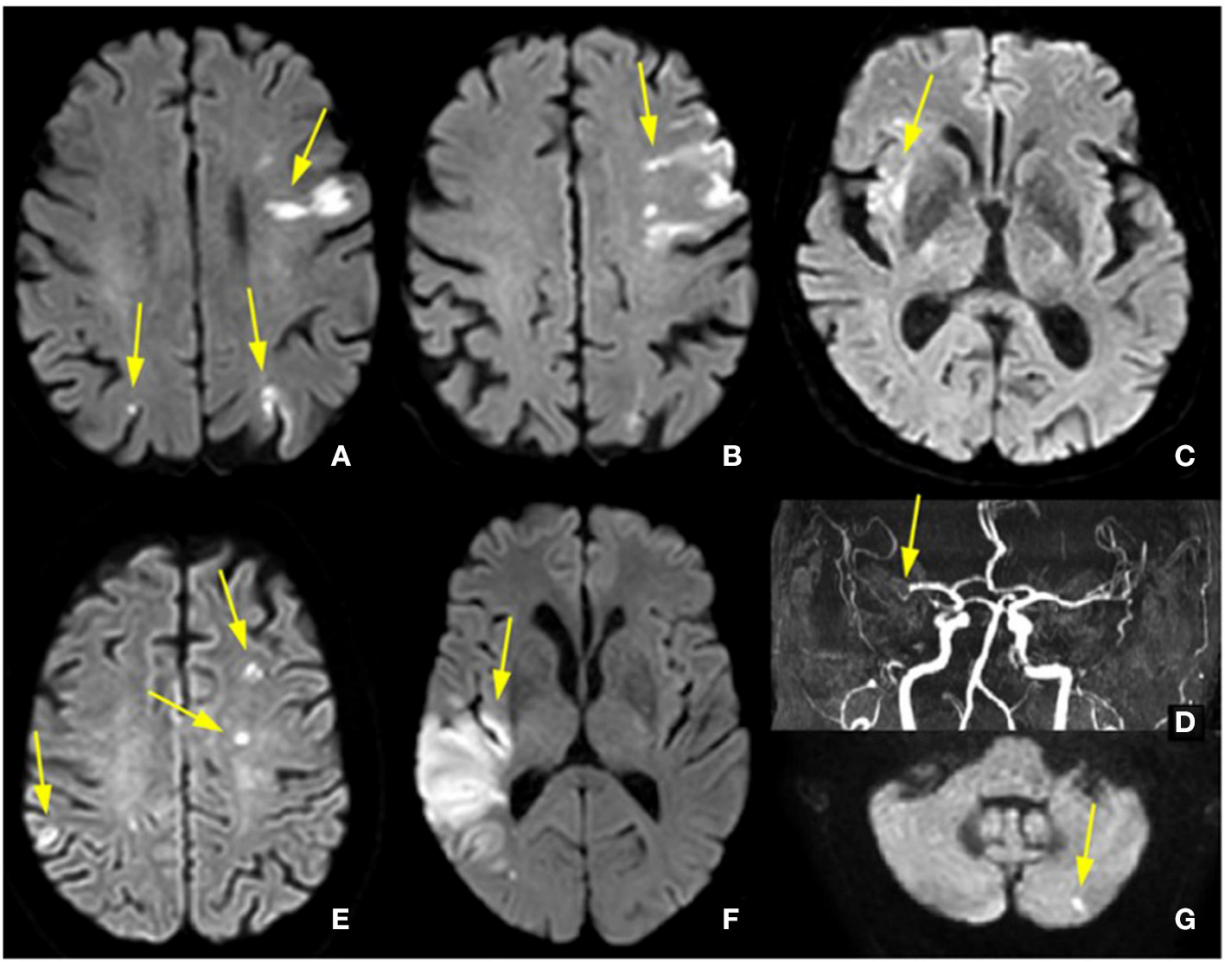
Micro- and macrovascular lesions with the involvement of multiple territories. **(A,B)** Diffusion-weighted images showing multiple bilateral ischemic lesions (arrows) in patient 4; **(C,D)** Diffusion-weighted images showing recent right insular infarct with thrombus in the right middle cerebral artery in patient 2; **(E-G)** Diffusion-weighted images showing multiple microvascular and macrovascular lesions in patient 6.

MRI revealed that more than one arterial territory was affected in four (44.4%) and four (8.2%) patients in the COVID-19 and control groups, respectively (*p* = 0.05). Additionally, regardless of the cause of stroke, four (44.4%) patients in the COVID-19 group and six (12.0%) patients in the control group had one or more microvascular infarcts (*p* = 0.04). In these four patients with COVID-19 and microvascular infarcts, macrovascular infarcts were observed in a different territory (Figure 2).

Mechanical thrombectomy was performed in one patient in the COVID-19 group (11.1%), and intravenous thrombolysis was performed in two (22.2%) and 10 (20.0%) patients in the COVID-19 and control groups, respectively, with combination therapy using both modalities in one patient in the COVID-19 group and in one patient in the control group. No death was observed attributable to the treatment in COVID-19 patients.

## Discussion

In this study, among the 772 COVID-19 patients hospitalized in our center, eight cases of ischemic stroke and one of TIA were diagnosed. No intracerebral hemorrhages (ICHs) were diagnosed. However, in the literature, ICHs are more less frequent than ischemic stroke (14). When comparing ischemic stroke characteristics between patients with COVID-19 and those without COVID-19 during the first 2 months of the COVID-19 outbreak, ischemic stroke in patients with COVID-19 appeared to be associated with more frequent multifocal territorial involvement, with more microvascular lesions, and more patients were positive for thrombophilia screening. The levels of inflammatory and coagulation markers (C-reactive protein, D-dimer, and fibrinogen) were higher in the COVID-19 group than those in the control group.

These results are different from those described by Yaghi et al. (15), who found that patients admitted during the same period to stroke centers in the New York metropolitan area with ischemic strokes associated with acute severe COVID-19 were younger (63 ± 17 vs. 70 ± 18, *p* = 0.001), had higher NIHSS scores (19 vs. 8, *p* = 0.007), and had more cryptogenic strokes (65.6 vs. 30.4%, *p* = 0.003) than were those with ischemic strokes in the absence of COVID-19. Szegedi et al. (16) reported a median NIHSS score of 16 in a cohort of 198 patients with acute COVID-19 who experienced stroke, including 23 patients with hemorrhagic stroke. In the present cohort of nine patients with COVID-19 and stroke, the mean NIHSS score was much lower (6.3) than that reported in previous studies with or without control groups (15, 16), and NIHSS score was similar in the two groups (6.3 ± 5.7 vs. 4.8 ± 5.8, *p* = 0.47). In the study by Yaghi et al. (15), 81.3% of the patients were admitted to the ICU due to severe SARS-CoV-2 infection, and 75.0% were dead or critically ill at last follow-up. The mortality rate was 64% in the study by Szegedi et al. (16), whereas only one patient (11.1%) had severe SARS-CoV-2 infection with acute respiratory distress syndrome justifying an ICU transfer, and none of the nine patients with COVID-19 died in the present study. This difference might partially explain the lower stroke rate in the present study. The severity of SARS-CoV-2 infection is a known risk factor for stroke (4, 16). The difference in the NIHSS score between the current study cohort and the previous studies might thus reflect differences in the severity of COVID-19.

Whereas, COVID-19 is known to cause severe cardiac injury and arrhythmias (8), which may lead to the formation of cardiac thrombi, in the present study, we did not observe a predominant, cardio-embolic, etiological mechanism of stroke in the COVID-19 group. An atherothrombotic mechanism, which might have been triggered by the inflammatory response to COVID-19 and an increased risk of plaque rupture, was not frequently found in our series.

Multifocal strokes, which have been described in patients with COVID-19 (17, 18), arise from a cardio-embolic source, arterio-arterial emboli from large arteries such as the aorta, carotid artery, and basilar artery, or circulating thrombi. In the present study, as in the population described by Yaghi et al. (15) the rate of cardio-embolic and atheromatous causes was the same in both groups.

Another possible explanation for the predominance of multifocal strokes in the present study is microvascular involvement in SARS-CoV-2 infection. In the present cohort, lesions smaller than 0.5 cm in size, indicating microvascular damage due to microthrombus, were more common in the COVID-19 group (*p* = 0.04). Autopsy (9, 19) and *in vivo* (20) studies suggest that COVID-19 is associated with endothelial injury and thrombotic microangiopathy. SARS-CoV-2 infects host cells via angiotensin-converting enzyme 2 receptors, which are expressed on endothelial cells, and the infection of endothelial cells leads to endothelial dysfunction (i.e., endothelial injury and lymphocytic endotheliitis) with microthrombus deposition and impaired microcirculatory function (9). Microvascular involvement could also be a consequence of hyperinflammation and neutrophil extracellular traps caused by COVID-19. These traps generate thrombin for fibrin production and represent part of a continuum of sterile inflammation and thrombosis that can involve all vascular beds, including the microvascular circulation (3). Elevated inflammatory parameters in patients with COVID-19-related stroke favor this explanation.

In the present study, the levels of coagulation markers fibrinogen and D-dimer were higher in the COVID-19 group than in the control group, similar to that observed in the study by Yaghi et al. (15), although the age and rates of high-risk conditions were similar between the two groups. The elevation in the levels of these markers may not only be related to the inflammatory response (21) but also reflect COVID-19-related coagulopathy, which overlaps with disseminated intravascular coagulation and thrombotic microangiopathy (3), thereby leading to the formation of multiple microthrombi and multifocal and microvascular infarcts. Inflammation markers, together with thrombophilia, should thus be considered as major determinants for the development of stroke in patients with COVID-19.

In the present study, the rate of thrombophilia was significantly higher in the COVID-19 group than in the control group. Beyrouti et al. reported that five of the six patients with COVID-19-related stroke in their cohort were positive for lupus anticoagulant; one of the patients also had anticardiolipin and anti-β2 glycoprotein-1 antibodies (16). These results must be taken with caution, since infection might have triggered the changes observed in these parameters (22, 23). Moreover, evidence that any of these markers is associated with stroke at all and that the numbers are far too small to draw any serious conclusions is lacking. But several other studies reported that COVID-19 was associated with antiphospholipid syndrome (24–26). The relationship between antiphospholipid antibodies in COVID-19 and COVID-19-associated coagulopathy remains controversial (27).

The present study has several limitations. This was a retrospective study with a limited number of cases. The incidence of acute ischemic stroke in the present study (1.16%) was lower than that reported in other reports (28). Another limitation is that for the control group, COVID-19 negativity was based on the absence of suggestive symptoms, negative RT-PCR, and serological results when performed (at a time when diagnoses were lacking). Adding new tests was not possible, so the possibility of asymptomatic COVID-19 in this group cannot be excluded. Infection severity is a risk factor for thrombosis (16). Moreover, especially at the beginning of pandemic, MRI or time-division multiplexing (TDM) were not systematically performed in patients in the ICU and those who were elderly, due to the rapidly deteriorating life-threatening conditions; thus, some events might have been missed among patients with very serious clinical conditions. The current study findings should be confirmed in prospective studies. However, our strength is having a controlled group, which allowed us to interpret the data more confidently.

## Conclusion

In the present study, we reported the different mechanisms of stroke in patients with COVID-19. Strokes affected several territories, with micro- and macro-vascular involvement. The main difference between the patients with and without COVID-19 who developed stroke was the presence of biological signs of hyperinflammation associated with thrombophilia. COVID-19-related strokes were frequently microvascular, suggesting endothelial inflammation and microthrombi due a procoagulant state as underlying causes. From a clinical standpoint, patients with COVID-19 should be considered to be at a higher risk for stroke. The low number of COVID-19 is, however, insufficient to draw robust conclusion, and larger cohorts are needed to confirm our results.

## Data Availability Statement

The original contributions presented in the study are included in the article/supplementary material, further inquiries can be directed to the corresponding author.

## Ethics Statement

The studies involving human participants were reviewed and approved by Comité d'Ethique des Facultés et Hôpitaux de Médecine, Odontologie et Pharmacie of the University Hospital of Strasbourg (approval no, CE-2020-32). Written informed consent for participation was not required for this study in accordance with the national legislation and the institutional requirements. Written informed consent was not obtained from the individual(s) for the publication of any potentially identifiable images or data included in this article.

## Author Contributions

GH, MM, and FS drafting/revising the manuscript for content, including medical writing for content, study concept or design, acquisition of data, analysis or interpretation of data, and study supervision and coordination. CS, M-HA-D, FV, and GA revising the manuscript for content and acquisition of data. CK revising the manuscript for content and statistical analysis and interpretation of data. All authors fulfill the criteria of authorship and no one else who fulfills the criteria has been excluded. All of them have approved the final submitted version.

## Conflict of Interest

The authors declare that the research was conducted in the absence of any commercial or financial relationships that could be construed as a potential conflict of interest.

## Publisher's Note

All claims expressed in this article are solely those of the authors and do not necessarily represent those of their affiliated organizations, or those of the publisher, the editors and the reviewers. Any product that may be evaluated in this article, or claim that may be made by its manufacturer, is not guaranteed or endorsed by the publisher.
